# Recent intimate partner violence among people with chronic mental illness: findings from a national cross-sectional survey

**DOI:** 10.1192/bjp.bp.114.144899

**Published:** 2015-09

**Authors:** Hind Khalifeh, Sian Oram, Kylee Trevillion, Sonia Johnson, Louise M. Howard

**Affiliations:** **Hind Khalifeh**, MSc MRCPsych, Mental Health Sciences Unit, Faculty of Brain Sciences, UCL, London; **Sian Oram**, PhD, **Kylee Trevillion**, PhD, Section of Women's Mental Health, Institute of Psychiatry, King's College London; **Sonia Johnson**, PhD, MRCPsych, Mental Health Sciences Unit, Faculty of Brain Sciences, UCL, London; **Louise M. Howard**, PhD, MRCPsych, Section of Women's Mental Health, Institute of Psychiatry, King's College London, UK

## Abstract

**Background**

People with mental illness are at increased risk of intimate partner violence (IPV) victimisation, but little is known about their risk for different forms of IPV, related health impact and help-seeking.

**Aims**

To estimate the odds for past-year IPV, related impact and disclosure among people with and without pre-existing chronic mental illness (CMI).

**Method**

We analysed data from 23 222 adult participants in the 2010/2011 British Crime Survey using multivariate logistic regression.

**Results**

Past-year IPV was reported by 21% and 10% of women and men with CMI, respectively. The adjusted relative odds for emotional, physical and sexual IPV among women with versus without CMI were 2.8 (CI = 1.9–4.0), 2.6 (CI = 1.6–4.3) and 5.4 (CI = 2.4–11.9), respectively. People with CMI were more likely to attempt suicide as result of IPV (aOR = 5.4, CI = 2.3–12.9), less likely to seek help from informal networks (aOR = 0.5, CI = 0.3–0.8) and more likely to seek help exclusively from health professionals (aOR = 6.9, CI = 2.6–18.3)

**Conclusions**

People with CMI are not only at increased risk of all forms of IPV, but they are more likely to suffer subsequent ill health and to disclose exclusively to health professionals. Therefore, health professionals play a key role in addressing IPV in this population.

Intimate partner violence (IPV) is associated with significant morbidity and mortality, especially among women, and its prevention is a global public health priority.^1^ IPV includes emotional abuse, threatening behaviour and physical or sexual violence between current and former partners.^1^ To date, research on IPV has predominantly focused on experiences of physical violence.^2^ In high-income settings, around 20% of women and 10% of men report lifetime IPV, with 5% and 3%, respectively, reporting past-year IPV.^3,4^ It is well established that IPV leads to mental health problems, including depression, post-traumatic stress disorder (PTSD) and suicide attempts.^5–7^ There is emerging evidence that this relationship is bidirectional – and that people (particularly women) with pre-existing mental disorders are at increased risk of subsequent IPV.^5,8,9^ However, little is known about their risk of different forms of IPV (especially emotional and sexual abuse), related health effects and help-seeking behaviour, or about the risk among men with mental disorders. Addressing this evidence gap is essential in guiding effective interventions in this vulnerable population.

Therefore, this study aims to examine the risk of recent IPV and related health burden and help-seeking behaviour among men and women with chronic (pre-existing) mental health problems in a nationally representative sample. We hypothesised that compared to people without chronic mental illness (CMI), people with CMI would be more likely to have experienced each type of IPV (emotional, physical and sexual IPV), and that victims with CMI would be (a) more likely to experience health problems and (b) less likely to seek help than victims without CMI.

## Method

### Data sources and study design

We used data from the 2010/2011 British Crime Survey (BCS).^10,11^ The BCS is an annual, nationally representative cross-sectional survey of crime victimisation in England and Wales. It is conducted by a private research company (BMRB), historically on behalf of the Home Office, and since 2012 on behalf of the Office for National Statistics. It comprises face-to-face interviews with all participants, and a self-completion module on domestic violence for participants aged 16–59 years only.

### Sampling, interview procedures and participants

The 2010/11 BCS recruited a random nationally representative sample of people aged 16 years or older living in private residential households in England and Wales. The sampling strategy was complex, and included stratification (in order to achieve a socio-demographically representative sample for each police force area) and clustering (for further details please see Home Office Technical Reports^10^). The widely used Postal Address File (the most complete record of private residential households) was used as the sampling frame. One adult was selected at random from each household, with no replacement in the case of non-participation.

Trained lay interviewers visited each selected household. Written informed consent was obtained from the selected adult after the study had been described. Each participant had a face-to-face computer-assisted interview conducted in a private setting in their home. This ‘main interview’ collected information on socio-demographics, past-year personal and household crime victimisation, and experiences of the criminal justice system. At the end of this interview, participants aged 16–59 years were additionally invited to consent to self-completion modules, which addressed domestic violence (including partner and family violence), sexual victimisation, stalking, drug-taking and drinking. These experiences were asked about in a self-completion questionnaire since they tend to be under-reported in face-to-face interviews.^12^ The participants were informed of the content and sensitive nature of the self-completion questionnaires, and re-assured of confidentiality. Participants could opt out of the self-completion modules if they were unwilling or unable to take part. Consenting participants were given a laptop and asked to read the questionnaires and enter the answers themselves, after which their answers were concealed. If they requested help from the interviewer with answering the self-completion module, questions on domestic and sexual violence were omitted.

In our study, we included all 2010/2011 BCS participants aged 16–59 years who completed the domestic violence module. We excluded those who had never had a partner, and those with missing data on partner violence (since partner violence was our primary outcome measure).

### Measurements

The main exposure was CMI. This was defined as ‘any long-standing mental health condition, such as depression’, which has lasted for 12 months or more and which limits day-to-day activities; as reported by participants in the face-to-face interview. No further details about the nature of CMI were available in this survey. Analysis of a separate national survey (the 2007 Adult Psychiatric Morbidity Survey), which used a similar CMI measure to that used in the BCS, found that 3.3% (*n* = 213) had self-reported CMI, of whom 81% (*n* = 173) had common mental disorders (depression or anxiety disorders) and 7.5% (*n* = 16) had probable psychosis or a diagnosis of psychosis; 75% had visited a GP and 20% had received mental health care from secondary mental health care services in the preceding year (further details available from author on request).

The main outcomes were: any past-year IPV-defined as any emotional, physical or sexual abuse by a current or former partner in the past 12 months; and the separate forms of IPV: emotional, physical and sexual. These were assessed by asking a series of questions on specific abusive behaviour, as detailed below.

#### IPV definition

1. Emotional abuse: partner did any of the following
  - Prevented them from having fair share of money
  - Stopped them from seeing friends or relatives
  - Repeatedly belittled them so they felt worthless
  - Threatened to hurt them or someone close to them
2. Physical abuse: partner did any of the following
  - Pushed them, held them down or slapped them
  - Kicked, bit or hit them, or threw something at them
  - Choked or tried to strangle them
  - Threatened them with a weapon or threatened to kill them
  - Used some other kind of force against them
3. Sexual abuse: partner did any of the following in a way that caused fear, alarm or distress
  - Indecently exposed themselves to them
  - Touched them sexually when they did not want it (e.g. groping, touching of breasts or bottom, unwanted kissing)
  - Sexually threatened them (e.g. demanded sex when they did not want it or followed or cornered them in a sexually threatening way)
  - Forced them to have sexual intercourse, or to take part in some other sexual act, when they made it clear that they did not agree or when they were not capable of consent

Since the definition of CMI for this study required a duration of more than 1 year, and the outcome of interest was IPV in the preceding year, the mental illness would by definition precede IPV (unless there was any measurement error).

Secondary outcomes were (a) health problems within the past 12 months ‘as a result of the abuse’, defined as: (i) physical illness or injury as a result of IPV (cuts/bruises/scratches/black eye/broken bones/internal injury/other physical injury/contracting a disease/becoming pregnant); (ii) mental/emotional problems as a result of IPV (difficulty sleeping/nightmares/depression/low self-esteem/attempted suicide); and (iii) attempted suicide as a result of IPV. (b) Help-seeking defined as telling one of the following three sources of help about IPV (i) informal network (family/relatives/friends/neighbours/work colleagues); (ii) health professionals (doctor/nurse/health visitor/counsellor/therapist); and (iii) other formal organisation (police/legal professional/government agency/helpline/specialist support service/voluntary organisation).

We estimated the prevalence and odds of each of the above outcomes among those with and without pre-existing CMI. We selected the following covariates as potential *a priori* confounders for any association between CMI and IPV, and between CMI and IPV-related health problems/help-seeking: gender, age, ethnicity (White/Black and minority ethnic), marital status (married/separated, divorced or widowed/ single), employment (employed, economically inactive, unemployed) and tenancy (homeowner, rents from private landlord, rents from local council).^12–14^ We additionally adjusted help-seeking for presence of adverse health outcomes as a result of IPV. It is worth noting that some of these covariates (e.g. marital status, employment, tenancy) may be conceptualised as either confounders or mediators. We conducted our multivariate analysis in two steps, first adjusting for age and gender, and then adjusting for the additional covariates of ethnicity, marital status, employment and tenancy, such that the relative contribution of these two sets of covariates to the strength of the association between CMI and IPV could be examined.

### Data analysis

We used the statistical software STATA version 12.0 SE (Stata Corporation, East College Station, TX, USA) for all analyses. We took the complex survey design into account (including clustering, stratification and population weights) using the STATA ‘svy’ suite commands. We estimated the population-weighted prevalence of IPV in those with and without CMI, and the prevalence standardised by age and gender (with the whole study sample as the reference population). Hypothesis tests were based on adjusted Pearson's tests (for bivariate analyses) or adjusted Wald tests (for multivariate logistic regression analyses). We tested for interactions between CMI and gender in relation to association with IPV. We stratified analyses for IPV prevalence and odds by gender.

## Results

### Response rate

The response rate for the 2010/11 BCS was 76% (*n* = 46 754 participants aged 16 years or more). Of those eligible for the questionnaire (*n* = 29 821 participants aged 16–59 years), 23 602 (79.1%) completed the module, 2297 (7.7%) refused and 3922 (13.2%) were unable to complete it without interviewer help (so they were not asked the domestic violence questions to maintain their safety).

**Figure 1:**
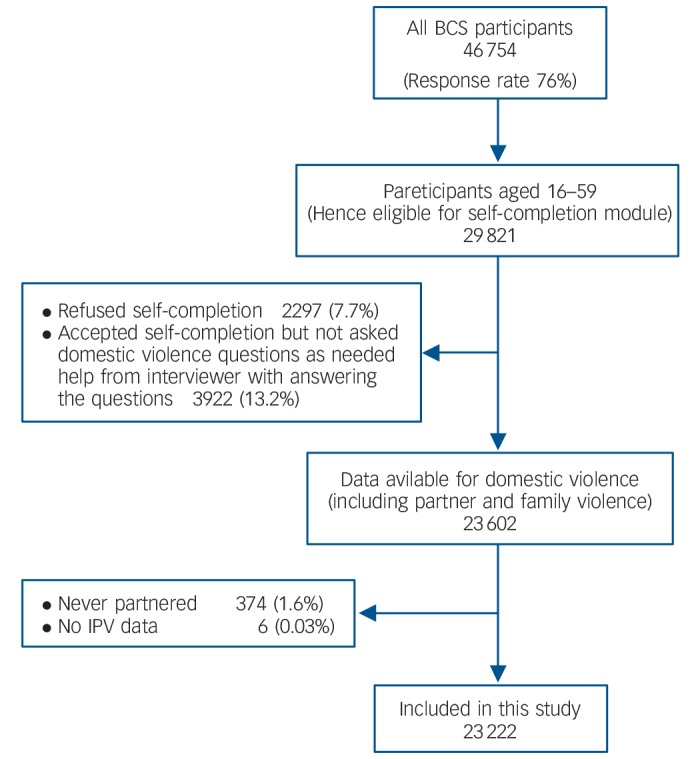
Sample flow

Of the 23 602 participants who completed the domestic violence module, we included 23 222 in our study sample after excluding 374 (1.6%) who never had a partner and six (0.03%) who had no data on partner violence. Completion of the domestic violence module was less likely amongst those with CMI (68% *v*. 80%), and among those who were older, from ethnic minorities or unemployed (Fig. 1).

### Prevalence of CMI and sample characteristics

The population-weighted prevalence of CMI was 2.4% (CI = 2.2%–2.7%; *n*/*N* = 692/23 222) with a prevalence of 2.7% (*n*/*N* = 442/12 731) and 2.1% (270/10 491) among women and men, respectively. Those with mental illness were more likely to be female, older, White, single, unemployed and to live in rented accommodation (see Table 1).

**Table 1:** Sample characteristics for those with and without mental illness

Characteristic^a^	Chronic mental illness *n* = 692, % (*n*)	No chronic mental illness *n* = 12 309, % (*n*)
Male	39.0 (270)	45.4 (10 221)
Female	61.0 (422)	54.6 (12 309)

White	94.1 (650)	91.3 (20 574)
Black and minority ethnic	5.9 (41)	8.7 (1950)
Missing	(1)	(6)

Married	35.7 (247)	58.6 (13 204)
Single	39.6 (274)	28.8 (6491)
Separated/divorced/widowed	24.7 (171)	12.6 (2828)

Employed	27.2 (188)	79.9 (17 957)
Economically inactive	64.6 (447)	15.7 (3523)
Unemployed	8.2 (57)	4.4 (991)
Missing	(0)	(41)

Owns house	32.6 (225)	64.7 (14 553)
Rents house from private landlord	26.5 (183)	21.7 (4881)
Rents house from Local Council	41.0 (283)	13.6 (3053)
Missing	(1)	(43)

Age: mean (s.d.)	40.6 (10.4)	39 (11.8)

a. All characteristics differed between groups at the 1% significance level.

### Prevalence and odds of past-year IPV

Among women, the population-weighted prevalence of past-year IPV was 20.0% (89/442) *v*. 5.3% (789/12 309) among those with and without CMI, respectively, and the age/gender standardised prevalence was 21.4% *v*. 5.6%, respectively (Table 2). Among men, the IPV population-weighted prevalence was 6.9% (21/271) *v*. 3.1% (356/10 221) among those with and without CMI, respectively, and the age/gender standardised prevalence was 10.1% *v*. 3.3%, respectively.

**Table 2:** Population-weighted and standardised prevalence of past-year IPV among those with and without chronic mental illness, by gender

	Men and women				Women				Men			
	Population-weighted prevalence (*n*)		Standardised prevalence (95% CI)^a^		Population-weighted prevalence (*n*)		Standardised prevalence (95% CI)^b^		Population-weighted prevalence (*n*)		Standardised prevalence (95% CI)^b^	
	CMI (*n* = 692)	No CMI (*n* = 22 530)	CMI	No CMI	CMI (*n* = 442)	No CMI (*n* = 12 309)	CMI	No CMI	CMI (*n* = 271)	No CMI (*n* = 10 221)	CMI	No CMI
Any IPV	14.6 (110)	4.3 (1145)	15.7 (11.4–20.1)	4.5 (4.1–4.8)	20.0 (89)	5.3 (789)	21.4 (16.1–26.7)	5.6 (5.1–6.1)	6.9 (21)	3.1 (356)	10.1 (3.4–16.7)	3.3 (2.9–3.8)

Emotional IPV	11.1 (90)	3.3 (897)	12.6 (8.6–16.6)	3.4 (3.1–3.7)	15.2 (73)	4.4 (653)	16.6 (12.2–21.1)	4.7 (4.2–5.2)	5.3 (17)	2.0 (244)	8.6 (2.2–15.0)	2.2 (1.8–2.5)

Physical IPV	6.1 (45)	1.8 (486)	7.1 (4.0–10.1)	1.8 (1.6–2.1)	8.1 (35)	2.2 (344)	9.9 (5.6–14.2)	2.5 (2.1–2.8)	3.2 (10)	1.2 (142)	4.3 (0.0–8.6)	1.2 (0.0–1.5)

Sexual IPV^c^	–	–	–	–	3.0 (13)	0.4 (68)	2.8 (1.3–4.4)	0.49 (0.34–0.64)	–	–	–	–

a. Standardised for age and gender.

b. Standardised for age.

c. Sexual IPV is reported for women only as the absolute numbers in men with mental illness were too low (<5) for stable estimates.

The adjusted OR for any IPV among people with CMI was 2.9 (CI = 2.1–3.8), with a trend for higher relative odds among women (OR = 3.3, CI = 2.4–4.7) than men (OR = 2.0, CI = 1.1–3.7) (interaction term for mental illnes×female gender = 1.8, CI = 0.97–3.5, *P* = 0.06; Table 3). Among women with CMI, the adjusted relative odds for emotional, physical and sexual IPV were 2.8 (CI = 1.9–4.0), 2.6 (CI = 1.6–4.3) and 5.4 (CI = 2.4–11.9), respectively. Among men with CMI, the adjusted relative odds for emotional and physical IPV were 2.0 (1.0–4.4) and 3.0 (1.2–7.5), respectively. The absolute number of men with CMI reporting sexual IPV was too small for stable estimates.

**Table 3:** Odds ratios for past-year IPV among people with and without chronic mental illness, by gender

	*n* among those with CMI	*n* among those without CMI	Age and gender-adjusted OR(95% CI)	*P*-value	Fully adjusted OR^a^ (95% CI)	*P*-value
Men and women	*n* = 692	*n* = 22 530				
Any IPV	110	1145	4.0 (3.0–5.2)	<0.001	2.9 (2.1–3.8)	<0.001
Emotional IPV	90	897	3.7 (2.8–5.0)	<0.001	2.5 (1.8–3.6)	<0.001
Physical IPV	45	486	3.7 (1.6–2.6)	<0.001	2.6 (1.7–4.0)	<0.001
Sexual IPV^b^	–	–	–		–	

Women	*n* = 442	*n* = 12 309				
Any IPV	89	789	4.7 (3.4–6.4)	<0.001	3.3 (2.4–4.7)	<0.001
Emotional IPV	73	653	4.0 (2.9–5.6)	<0.001	2.8 (1.9–4.0)	<0.001
Physical IPV	35	344	4.0 (2.4–6.7)	<0.001	2.6 (1.6–4.3)	<0.001
Sexual IPV	13	68	8.0 (4.1–15.6)	<0.001	5.4 (2.4–11.9)	<0.001

Men	*n* = 270	*n* = 10 221				
Any IPV	21	356	2.61 (1.5–4.6)	<0.01	2.0 (1.1–3.7)	0.03
Emotional IPV	17	244	2.9 (1.5–5.5)	<0.01	2.0 (1.0–4.4)	0.04
Physical IPV	10	142	3.0 (1.4–6.4)	<0.01	3.0 (1.2–7.5)	0.02
Sexual IPV^b^	–	–	–		–	

a. Final model included age, gender, ethnicity, marital status, housing tenure, employment status.

b. Sexual IPV is reported for women only, as the absolute numbers in men with MI is too small (<5) for stable estimates.

### Health problems among IPV victims

Comparing health problems for victims with and without pre-existing CMI, the former were more likely to experience emotional/mental problems within the past year as a result of IPV (53% *v*. 30%; OR adjusted for socio-demographics = 2.2, CI = 1.3–3.8), with particularly high relative odds for attempted suicide as a result of IPV (13% *v*. 2%, aOR = 5.4, CI = 2.3–12.9; Table 4). The two groups were equally likely to experience physical injuries/illness following IPV (24%, *P* = 0.97).

**Table 4:** Prevalence and odds of health problems as a result of IPV among victims with and without chronic mental illness

Problems as a result of IPV	Victims with CMI *n* = 109 % (*n*)	Victims without CMI *n* = 1142 % (*n*)	OR adjusted for age and gender (95% CI)	Fully adjusted OR (95% CI)^a^	*P* for fully adjusted OR
Any health problems	57.9 (64)	41.3 (493)	1.9 (1.1–3.1)	1.8 (1.1–3.0)	0.02

Physical injury/illness	23.9 (23)	23.7 (283)	1.0 (0.5–2.0)	0.9 (0.5–1.8)	0.75

Mental/emotional problems	53.2 (60)	30.5 (359)	2.4 (1.5–4.0)	2.2 (1.3–3.8)	<0.01

Suicide attempts	12.8 (14)	2.2 (25)	4.9 (2.1–11.1)	5.4 (2.3–12.9)	<0.001

a. Adjusted for age, gender, ethnicity, marital status, employment, tenancy.

### Help-seeking among IPV victims

Victims with and without CMI were equally likely to seek help from any source; 52% *v*. 51% among all victims (*P* = 0.91) and 69% *v*. 78% among victims who experienced health problems as a result of the abuse (*P* = 0.06; Table 5). However, victims with CMI were less likely to seek help from informal networks (OR adjusted for socio-demographics and health problems = 0.47, CI = 0.27–0.83) and more likely to seek help from health professionals (aOR = 2.7, CI = 1.3–5.1) than victims without CMI. Most victims sought help from more than one source, but 12% of victims with CMI (*v*. 1.5% of those without) sought help exclusively from health professionals (aOR = 6.9, CI = 2.6–18.3).

**Table 5:** Prevalence and odds of disclosure of IPV by victims with and without chronic mental illness

Disclosed IPV to:	IPV victims with or without health problems as a result of IPV		IPV victims with health problems as a result of IPV		OR adjusted for age and gender (95% CI)	Fully adjusted OR (95% CI)^a^	*P* for fully adjusted OR
	CMI *n* = 110 % (*n*)	No CMI *n* = 1145 % (*n*)	CMI *n* = 64 % (*n*)	No CMI = 493 % (*n*)			
Anyone	52.4 (57)	50.8 (601)	68.9 (44)	77.8 (390)	1.0 (0.6–1.6)	0.6 (0.4–1.2)	0.15

Informal	37.0 (38)	45.0 (521)	52.0 (31)	70.4 (343)	0.68 (0.40–1.1)	0.5 (0.3–0.8)	<0.01

Health professionals	35.0 (42)	13.4 (171)	44.3 (34)	25.6 (138)	3.2 (1.9–5.5)	2.7 (1.3–5.5)	<0.01

Other formal	23.3 (28)	17.8 (231)	39.0 (26)	33.2 (174)	1.2 (0.60–2.3)	0.7 (0.4–1.3)	0.27

HP only	11.8 (12)	1.5 (24)	12.5 (8)	3.1 (20)	7.0 (3.0–16.2)	6.9 (2.6–18.3)	<0.001

a. Adjusted for age, gender, ethnicity, marital status, employment, tenancy and presence of health problems as a result of IPV.

## Discussion

### Key findings

In a large, nationally representative crime survey in England and Wales, and comparing people with and without CMI of more than 1 year's duration; the population-weighted prevalence of being a victim of any IPV in the past year was 20.0% *v*. 5.3% among women and 6.9% *v*. 3.1% among men. After adjusting for socio-demographics, we found that people with CMI were two to five times more likely to experience emotional, physical and sexual IPV as those without (with a trend for higher odds among women than men). The highest relative odds were found for sexual IPV against women; 30 in 1000 women with CMI reported sexual assault by a partner in the past year compared to 4 in 1000 of women without CMI (with adjusted relative odds of 5.4). Victims with CMI were twice as likely to experience mental or emotional problems and five times more likely to attempt suicide as a result of this IPV than victims without CMI. Whilst there was no difference in overall disclosure rates between victims with and without CMI, the former were half as likely disclose IPV to informal social networks, but twice as likely to disclose it to a health professional. Most victims disclosed their experiences of IPV to multiple parties, but around 10% of IPV victims with CMI (and 1.5% of those without) disclosed exclusively to a health professional.

### Strengths and limitations

The strengths of this study include clearly defined hypotheses; a large adequately powered study; a nationally representative sample; and detailed measures of IPV impact and disclosure. The IPV measure used was one that was specifically developed for use in the BCS^12^ and has strengths and limitations. Its strengths include two features that are widely recognised as good practice in IPV/domestic violence research: (a) the use of specific behavioural questions on whether the respondent had experienced specific acts of violence over a well-defined time period, rather than more generic questions on whether they had experienced ‘abuse’ or ‘domestic violence; (b) the use of a self-completion questionnaire rather than interview-based questionnaire, which provides greater privacy, confidentiality and safety for the respondent.^3^ Both of these features are associated with greater rates of disclosure,^3^ with past Home Office research showing that respondents were five times more likely to disclose domestic violence when asked about this in the self-completion questionnaire than when asked about it in the interviewer-based questionnaire.^12^ Limitations of this IPV measure include (a) the lack of validation studies against other ‘gold standard’ IPV measures and (b) the lack of sufficient detail on the context, nature and frequency of violence which would allow a distinction between isolated acts of violence *v.* prolonged, severe and controlling violence.^15^ Other strengths of this study include adjustment for important socio-demographic confounders. We carried out additional analyses on a separate national survey (the 2007 Adult Psychiatric Morbidity Survey-APMS), which used a similar CMI measure to that used in the BCS,^16^ and which enabled us to indirectly assess the potential clinical characteristics of this group.

Study limitations include lack of details on the nature of the CMI within the BCS dataset, although the study definition meets internationally accepted definitions of disability,^17^ and we indirectly assessed clinical characteristics of people with CMI using APMS data. There is potential for non-participation bias, since nearly 20% (*N* = 6219) of those eligible for the self-completion questionnaire did not complete the domestic violence module, either because they refused (8%) or because they were unable to complete the module without interviewer help (13%). Non-respondents were more likely to be older, unemployed and from an ethnic minority background. Some of these factors are associated with IPV risk, and so may lead to bias in the prevalence estimates. The domestic violence module was also less likely to be completed by those with versus those without CMI. Those with the greatest disability may be more likely to opt out of participating in the crime survey, and where they do participate they are more likely to opt out of the optional domestic violence module (since it increases participant burden). Greater disability is likely to be associated with greater IPV risk; therefore, we may have underestimated the relative risk for those with mental illness. It is possible that people with and without mental illness had differential recall or reporting of IPV, although there is some evidence that self-reported victimisation among people with mental illness is reliable.^18^ A limitation of cross-sectional studies is that it is often difficult to be certain of the direction of causality. In this study, the definitions of ‘CMI’ (duration more than 1 year) and ‘recent IPV’ (within the past year) mean that mental illness would have preceded ‘recent IPV’, except where there was measurement error due to reporting or recall bias. Nonetheless, the onset of IPV and CMI was not measured, and some participants may have experienced IPV before the onset of their mental illness (where IPV may have causally contributed to their CMI). The association found in this study between CMI and IPV could plausibly explained by two pathways: mental illness could be a risk factor for IPV and/or historical IPV could be a risk factor for both CMI and recent IPV. Both of these pathways are supported by a recent systematic review of longitudinal studies, which found evidence for a bidirectional causal relationship between common mental disorders and IPV.^19^ Findings are likely to generalise to other high-income settings, which tend to have similar prevalence and risk factors for IPV.^3^

### Findings in the context of past studies and implications

This is the first study to directly compare recent IPV (emotional, physical and sexual) among men and women with and without pre-existing mental illness.^5,8,9,20^ Our findings on the prevalence and relative odds of any past-year IPV among women with mental illness are consistent with recent systematic reviews.^5,8,20^ We extend previous findings by reporting an excess risk in men as well as in women with mental illness; and an excess risk for all forms of IPV, including emotional and sexual IPV. The findings on emotional IPV are important, since there is evidence that emotional IPV may lead to greater health problems than physical IPV,^21–23^ whereas most research and clinical enquiry is focused on the latter. We found that the greatest relative odds were for sexual IPV against women, which were increased fivefold, with 3 in 100 women with CMI reporting past-year sexual violence by a partner. These findings suggest that health professionals should enquire about all forms of recent IPV, including emotional and sexual abuse, among people with CMI.

We report the novel finding that victims with pre-existing mental illness had an excess risk of psychological ill health (including suicide attempts) following IPV; consistent with findings on the greater psychological health impact of community violence against people with mental illness.^24^ We also found that they were more likely to disclose IPV exclusively to healthcare professionals. These findings underline the key role that health professionals play in detecting IPV and supporting victims amongst this vulnerable population. The APMS analysis suggests that the majority of people with self-reported CMI have sought help from primary care, and about 20% have sought help from secondary health services in the preceding year, providing an opportunity for interventions by healthcare professionals. However, IPV is under-detected by primary care and mental health professionals,^25,26^ who report a lack of knowledge and preparedness to address IPV.^27,28^

Complex interventions that include staff training and integration of advocacy workers within healthcare settings have been shown to improve detection of IPV and subsequent referral.^29,30^ Recent NICE guidance on domestic violence^31^ emphasises that identification of, and appropriate responses to, IPV among mental health service users should be part of good clinical practice; so mental health professionals should be trained to respond safely within well-defined care pathways.^32^ This study found that women with CMI were particularly vulnerable to sexual violence, suggesting that training for mental health professionals should be tailored accordingly. However, there are still few studies on specific interventions for IPV in people with CMI.^33^ Future research should focus on interventions to decrease the risk and impact of IPV among those with mental illness.

## References

[1] World Health Organisation Responding to Intimate Partner and Sexual Violence Against Women: WHO Clinical and Policy Guidelines. WHO, 2013. 24354041

[2] Feder G Ramsay J Dunne D Rose M Arsene C Norman R How far does screening women for domestic (partner) violence in different healthcare settings meet criteria for a screening programme? Systematic reviews of nine UK National Screening Committee criteria. Health Technol Assess 2009; 13(16): iii–iv, xi–xiii, 1–113, 137–347. 19272272 10.3310/hta13160

[3] World Health Organisation Global and Regional Estimates of Violence Against Women: Prevalence and Health Effects of Intimate Partner Violence and Non-partner Sexual Violence. WHO, 2013.

[4] Smith K Osborne S Lau I Britton A Homicides, Firearm Offences and Intimate Violence 2010/11: Supplementary Volume 2 to Crime in England and Wales 2010/11. Home Office, 2012.

[5] Devries KM Mak JY Bacchus LJ Child JC Falder G Petzold M Intimate partner violence and incident depressive symptoms and suicide attempts: a systematic review of longitudinal studies. PLoS Med 2013; 10: 11. 10.1371/journal.pmed.1001439 PMC3646718 23671407

[6] Coker AL Davis KE Arias I Desai S Sanderson M Brandt HM Physical and mental health effects of intimate partner violence for men and women. Am J Preventive Med 2002; 23: 260–8. 10.1016/s0749-3797(02)00514-7 12406480

[7] Afifi TO MacMillan H Cox BJ Asmundson GJG Stein MB Sareen J Mental health correlates of intimate partner violence in marital relationships in a nationally representative sample of males and females. J Interpersonal Violence 2009; 24: 1398–417. 10.1177/0886260508322192 18718882

[8] Trevillion K Oram S Feder G Howard LM Experiences of domestic violence and mental disorders: a systematic review and meta-analysis. PLoS ONE 2012; 7: e51740. 23300562 10.1371/journal.pone.0051740 PMC3530507

[9] Oram S Trevillion K Feder G Howard LM Prevalence of experiences of domestic violence among psychiatric patients: systematic review. Br J Psychiatry 2013; 202: 94–9. 23377208 10.1192/bjp.bp.112.109934

[10] Fitzpatrick A Grant C The 2010/11 British Crime Survey (England and Wales): Technical Report Vol. 1 TNS-BMRB, 2011.

[11] Chaplin R Flatley J Smith K Crime in England and Wales 2010/11: Findings from the British Crime Survey and Police Recorded Crime 2nd edn. Home Office, 2011.

[12] Walby S Allen J Domestic Violence, Sexual Assault and Stalking: Findings from the British Crime Survey. Home Office, 2004.

[13] Capaldi DM Knoble NB Shortt JW Kim HK A systematic review of risk factors for intimate partner violence. Partner Abuse 2012; 3: 231–80. 22754606 10.1891/1946-6560.3.2.231 PMC3384540

[14] Khalifeh H Hargreaves J Howard LM Birdthistle I Intimate partner violence and socioeconomic deprivation in England: findings from a national cross-sectional survey. Am J Public Health 2013; 103: 462–72. 22897532 10.2105/AJPH.2012.300723 PMC3673488

[15] Johnson MP Conflict and control – gender symmetry and asymmetry in domestic violence. Violence Against Women 2006; 12: 1003–18. 17043363 10.1177/1077801206293328

[16] McManus S Meltzer H Brugha T Bebbington P Jenkins R Adult Psychiatric Morbidity in England, 2007: Results of a Household Survey. The NHS Information Centre, 2010.

[17] World Health Organisation World Report on Disability. WHO, 2011.

[18] Goodman LA Thompson KM Weinfurt K Corl S Acker P Mueser KT Reliability of reports of violent victimization and posttraumatic stress disorder among men and women with serious mental illness. J Traumatic Stress 1999; 12: 587–99. 10.1023/A:1024708916143 10646178

[19] Devries KM Mak JY Bacchus LJ Child JC Falder G Petzold M Intimate partner violence and incident depressive symptoms and suicide attempts: a systematic review of longitudinal studies. PLoS Med 2013; 10: e1001439. 23671407 10.1371/journal.pmed.1001439 PMC3646718

[20] Hughes K Bellis MA Jones L Wood S Bates G Eckley L Prevalence and risk of violence against adults with disabilities: a systematic review and meta-analysis of observational studies. Lancet 2012; 379: 1621–9. 22377290 10.1016/S0140-6736(11)61851-5

[21] Jewkes R Emotional abuse: a neglected dimension of partner violence. Lancet 2010; 376: 851–2. 20822808 10.1016/S0140-6736(10)61079-3

[22] Yoshihama M Horrocks J Kamano S The role of emotional abuse in intimate partner violence and health among women in Yokohama, Japan. Am J Public Health, 2009; 99: 647–53. 18703455 10.2105/AJPH.2007.118976 PMC2661493

[23] Mechanic MB Weaver TL Resick PA Mental health consequences of intimate partner abuse – a multidimensional assessment of four different forms of abuse. Violence Against Women 2008; 14: 634–54. 18535306 10.1177/1077801208319283 PMC2967430

[24] Khalifeh H Howard LM Osborn D Moran P Johnson S Violence against people with disability in England and Wales: findings from a national cross-sectional survey. PLoS ONE 2013; 8: e55952. 23437079 10.1371/journal.pone.0055952 PMC3577814

[25] Howard L Trevillion K Khalifeh H Woodall A Agnew-Davies R Feder G Domestic violence and severe psychiatric disorders: prevalence and interventions. Psychological Med 2010; 40: 881–93. 10.1017/S0033291709991589 19891808

[26] Ramsay J Rutterford C Gregory A Dunne D Eldridge S Sharp D Domestic violence: knowledge, attitudes, and clinical practice of selected UK primary healthcare clinicians. Br J Gen Pract 2012; 62: e647–55. 22947586 10.3399/bjgp12X654623 PMC3426604

[27] Rose D Trevillion K Woodall A Morgan C Feder G Howard L Barriers and facilitators of disclosures of domestic violence by mental health service users: qualitative study. Br J Psychiatry 2011; 198: 189–94. 21160053 10.1192/bjp.bp.109.072389

[28] Nyame S Howard LM Feder G Trevillion K A survey of mental health professionals' knowledge, attitudes and preparedness to respond to domestic violence. J Mental Health 2013; 22: 536–43. 10.3109/09638237.2013.841871 24279406

[29] Feder G Davies RA Baird K Dunne D Eldridge S Griffiths C Identification and referral to improve safety (IRIS) of women experiencing domestic violence with a primary care training and support programme: a cluster randomised controlled trial. Lancet 2011; 378: 1788–95. 22000683 10.1016/S0140-6736(11)61179-3

[30] Trevillion K Byford S Cary M Rose D Oram S Feder G Linking abuse and recovery through advocacy: an observational study. Epidemiol Psychiatr Sci 2014; 23: 99–113. 23628450 10.1017/S2045796013000206 PMC6998308

[31] NICE Domestic Violence and Abuse – Identification and Prevention: Draft Guidance. NICE, 2013.

[32] Howard LM Feder G Agnew-Davies R Domestic Violence and Mental Health. RCPsych Publications, 2013.

[33] Warshaw C Sullivan C Rivera E A Systematic Review of Trauma-Focused Interventions for Domestic Violence Survivors. National Collaborating Center for Domestic Violence, Trauma and Mental Health, 2013.

